# Reliability of the Hazelbaker Assessment Tool for Lingual Frenulum Function

**DOI:** 10.1186/1746-4358-1-3

**Published:** 2006-03-09

**Authors:** Lisa H Amir, Jennifer P James, Susan M Donath

**Affiliations:** 1 Key Centre for Women's Health in Society, University of Melbourne, Australia; 2 Breastfeeding Education and Support Services, The Royal Women's Hospital, Melbourne, Australia; 3 Clinical Epidemiology and Biostatistics Unit, Murdoch Children's Research Institute, Royal Children's Hospital, Melbourne, Australia

## Abstract

**Background:**

About 3% of infants are born with a tongue-tie which may lead to breastfeeding problems such as ineffective latch, painful attachment or poor weight gain. The Hazelbaker Assessment Tool for Lingual Frenulum Function (HATLFF) has been developed to give a quantitative assessment of the tongue-tie and recommendation about frenotomy (release of the frenulum). The aim of this study was to assess the inter-rater reliability of the HATLFF.

**Methods:**

Fifty-eight infants referred to the Breastfeeding Education and Support Services (BESS) at The Royal Women's Hospital for assessment of tongue-tie and 25 control infants were assessed by two clinicians independently.

**Results:**

The Appearance items received kappas between about 0.4 to 0.6, which represents "moderate" reliability. The first three Function items (lateralization, lift and extension of tongue) had kappa values over 0.65 which indicates "substantial" agreement. The four Function items relating to infant sucking (spread, cupping, peristalsis and snapback) received low kappa values with insignificant p values. There was 96% agreement between the two assessors on the recommendation for frenotomy (kappa 0.92, excellent agreement). The study found that the Function Score can be more simply assessed using only the first three function items (ie not scoring the sucking items), with a cut-off of ≤4 for recommendation of frenotomy.

**Conclusion:**

We found that the HATLFF has a high reliability in a study of infants with tongue-tie and control infants

## Background

About 3% of infants are born with a tongue-tie or partial ankyloglossia [1]. The Academy of Breastfeeding Medicine Protocol defines partial ankyloglossia as "the presence of a sublingual frenulum which changes the appearance and/or function of the infant's tongue because of its decreased length, lack of elasticity or attachment too distal beneath the tongue or too close to or onto the gingival ridge" [[2] p1]. Complete ankyloglossia in which there is extensive fusion of the tongue to the floor of the mouth is extremely rare.

Since the early 1990s, a number of case studies and case series of infants with tongue-tie experiencing feeding problems, such as ineffective latch, painful attachment and poor weight gain, have been published in the breastfeeding literature [3-6]. Many of the case studies/series report an improved ability to breastfeed following a simple release of the frenulum (frenotomy) [7]. In infants aged up to about three months, clinicians have found that it is safe to release the frenulum with a small pair of sterile scissors when the infant having difficulty breastfeeding is found to have a tight frenulum comprised of a thin membrane [7]. Our practice has been to release the frenulum in infants if there is a significant tongue-tie and there is evidence of difficulty breastfeeding such as slow rate of milk transfer or ongoing nipple pain or trauma.

A range of clinicians perform this simple frenotomy: dentists, surgeons, paediatricians, obstetricians, general practitioners and ear, nose and throat specialists [3,7]. In some settings, infant feeding specialists are also performing frenotomies after appropriate training [8,9], however this does raise "legal and ethical issues about the scope of lactation consultant practice" which varies around the world [10] p413].

Our review of 35 infants following tongue-tie release found a high level of parental satisfaction and no complications [11]. Some of the parents reported that they appreciated the careful examination of the infant's mouth during the assessment procedure. For example one parent stated "Very pleased with assessment" [11] p245].

Correct attachment to the breast involves the infant moving the tongue forward to grasp and draw the nipple and surrounding breast tissue well into the mouth to form a teat [12,13]. Some infants with tongue-tie are unable to grasp the nipple/breast, while others attach poorly causing nipple pain or damage [2]. The aetiology of breastfeeding difficulties in these infants has not been elucidated, however, ultrasound studies may provide some evidence in the future. A preliminary study of ten infants using submental ultrasound assessment detected a change in nipple position and tongue movement during a feed following frenotomy [14].

An RCT in Southampton, UK, in 2002 identified infants with a tongue-tie who were experiencing breastfeeding problems [8]. Fifty-seven infants were randomly assigned to have immediate frenotomy by the lactation consultant/infant feeding specialist or to receive help with positioning and attachment by the lactation consultant and review in 48 hours. They found that releasing the tongue-tie improved feeding in 27 out of 28 infants, compared to 1 out of 29 who improved without release [8].

As there is no generally agreed definition of tongue-tie, a quantitative instrument has been developed: the Hazelbaker Assessment Tool for Lingual Frenulum Function (HATLFF) [15]. Alison Hazelbaker stated "Because of my personal experiences with ankyloglossia and my frustration with the lack of a formal way to assess its presence in breastfed infants, I wanted to develop an assessment approach that would make it easier to determine the extent of the impact that tongue-tie had on tongue mobility in the breastfed baby" [15] p47]. Five appearance items, such as length of lingual frenulum (>1 cm, 1 cm, <1 cm) and seven function items, such as extension of the tongue (tip over the lower lip, tip over lower gum only, neither) are assessed (See Table 1). Ballard and colleagues have explained how to score each item [1]. Hazelbaker has demonstrated that the tool has content validity, however, it needs to be formally assessed for reliability [1], and this was the aim of this study.

**Figure 1:**
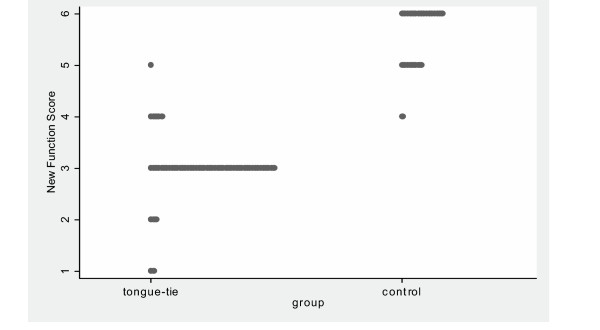
Dot-plot of new Function Score.

**Table 1:** Hazelbaker Assessment Tool for Lingual Frenulum Function (1998 version)

**Appearance Items**
Appearance of tongue when lifted
2: Round OR square
1: Slight cleft in tip apparent
0: Heart-shaped
Elasticity of frenulum
2: Very elastic (excellent)
1: Moderately elastic
0: Little OR no elasticity
Length of lingual frenulum when tongue lifted
2: More than 1 cm OR embedded in tongue
1: 1 cm
0: Less than 1 cm
Attachment of lingual frenulum to tongue:
2: Posterior to tip
1: At tip
0: Notched tip
Attachment of lingual frenulum to inferior alveolar ridge
2: Attached to floor of mouth OR well below ridge
1: Attached just below ridge
0: Attached at ridge

**Function Items**

Lateralization
2: Complete
1: Body of tongue but not tongue tip
0: None
Lift of tongue
2: Tip to mid-mouth
1: Only edges to mid-mouth
0: Tip stays at alveolar ridge or rises to mid-mouth only with jaw closure
Extension of tongue:
2: Tip over lower lip
1: Tip over lower gum only
0: Neither of above, OR anterior or mid-tongue humps
Spread of anterior tongue
2: Complete
1: Moderate OR partial
0: Little OR none
Cupping
2: Entire edge, firm cup
1: Side edges only, moderate cup
0: Poor OR no cup
Peristalsis:
2: Complete, anterior to posterior (originates at the tip)
1: Partial: originating posterior to tip
0: None OR reverse peristalsis
Snapback
2: None
1: Periodic
0: Frequent OR with each suck

^© ^Alison K. Hazelbaker, MA, IBCLC July 1 1998

14 = Perfect score (regardless of Appearance Item score)

11 = Acceptable if Appearance Item score is 10

<11 = Function impaired. Frenotomy should be considered if management fails. Frenotomy necessary if Appearance Item score is <8.

## Method

The primary hypotheses were that:

1. Two assessors will give infants referred for tongue-tie similar recommendations for release based on the Hazelbaker Assessment Tool for Lingual Frenulum with an inter-rater reliability of at least 0.75 (kappa).

2. Two assessors will give normal infants similar recommendations based on the Hazelbaker Assessment Tool for Lingual Frenulum with an inter-rater reliability of at least 0.6 (kappa).

Infants referred to the Breastfeeding Education and Support Service (BESS) at The Royal Women's Hospital for assessment of tongue-tie were assessed by the BESS medical practitioner (LHA) and a second assessor, one of the BESS lactation consultants (usually JPJ). A convenience sample of healthy infants attending BESS was also assessed by two assessors. Parents were informed about the study, given a Plain Language Statement and signed the Informed Consent form before participating.

Basic demographic information was collected, including age of mother and baby, parity, mother's level of education and private health insurance. Information about breastfeeding and family history of tongue-tie was also collected.

The infants were assessed by two assessors, who each completed the HATLFF independently prior to a feed.

Inter-rater reliability was measured using kappa for agreement between the assessors on recommendation for tongue-tie release. Kappa is a measurement of the proportion of potential agreement beyond chance (actual agreement beyond chance/potential agreement beyond chance) [16]. Some experts have described the value of kappa as 0 to 0.2 as "slight", 0.2 to 0.4 as "fair", 0.4 to 0.6 as "moderate", 0.6 to 0.8 as "substantial" and 0.8 to 1.0 as "almost perfect" [16]. In most clinical examinations, agreement between examiners is not perfect, and a kappa of 0.4 is common [16]. Cronbach's alpha was used to examine the correlation between the items. Cronbach's alpha measures the inter-item correlations, ie how closely the items fit together to describe something. It is recommended that the value of alpha should be between 0.70 and 0.90 [17]. In the group of infants assessed for tongue-tie, we expected approximately 75% of infants to be scored as recommending frenulum release (from previous study) [11]. Assuming a kappa of 0.75 with a precision of 0.2, the sample size would be 48 infants. Sample size calculated using Stata 8.0 (sskdlg procedure). Therefore we planned to recruit 50 infants with tongue-tie and 50 control infants.

The study was approved by The Royal Women's Hospital Research and Ethics Committee (04/24, 7 Sept 2004) and the University of Melbourne (HREC 040676, 17 Sept 2004).

## Results

Infants were recruited from September 2004 to April 2005. Fifty-eight infants with tongue-tie were recruited, age range 1 day to 84 days, mean 17 days, median 10 days, 56% were male (32/57, 1 missing). There were 25 control infants age with an age range from 7–55 days, mean 26, median 22 days.

Table 2 shows the reliability of each item in the Assessment Tool. The Appearance items received kappas between about 0.4 to 0.6, which represents "moderate" reliability. The first three Function items (lateralization, lift and extension of tongue) had kappa values over 0.65 which indicates "substantial" agreement. The four Function items relating to infant sucking (spread, cupping, peristalsis and snapback) received low kappa values with insignificant p values.

**Table 2:** Reliability of each item

**Item**	**Kappa**	**P value**
**Appearance items**		
Appearance of tongue when lifted	0.54	<0.01
Elasticity of frenulum	0.53	<0.01
Length of lingual frenulum when tongue lifted	0.51	<0.01
Attachment of lingual frenulum to tongue	0.39	<0.01
Attachment of lingual frenulum to inferior alveolar ridge	0.62	<0.01
**Function items**		
Lateralization	0.71	<0.01
Lift of tongue	0.67	<0.01
Extension of tongue	0.65	<0.01
Spread of anterior tongue	-0.02	0.74
Cupping	0.01	0.44
Peristalsis	0.05	0.07
Snapback	0.03	0.38

There was 96% agreement between the two assessors on the recommendation for frenotomy (see Table 3). The kappa statistic was 0.92, which represents excellent agreement or "almost perfect" [16].

**Table 3:** Assessors' recommendations.

	**Recommendation – Assessor 2**		
	
**Recommendation – Assessor 1**	Release	No need to release	Total
Release	56	0	56
No need to release	3	24	27
Total	59	24	83

The items in each part of the assessment tool were examined to see how well the items fitted together. For the seven Function items Cronbach's alpha was 0.5074 (ie low). When the four items relating to the infant's sucking were removed (spread, cupping, peristalsis and snap-back), the three items remaining in the new Function Score received a higher reliability with a Cronbach's alpha of 0.8655. The five items contributing to the Appearance Score had a Cronbach's alpha of 0.7487. This is an acceptable reliability and did not alter if individual items were dropped.

The dot-plot (Figure) shows the distribution of new Function scores: from 0 to 6. Most of the infants referred with tongue-tie scored 4 or less, while most of the control infants scored 5 or 6. Therefore a cut-off of "less than or equal to 4" indicating a recommendation for frenotomy was chosen for the new Function score. This new cut-off has a high sensitivity and specificity with an area under the ROC curve of 0.9948.

There was no difference in any recommendation (ie whether to release or not) between the old Function score and the new Function score. Therefore, there was no change in the kappa statistic with the new shorter Function score: 0.92 (excellent agreement).

## Discussion and conclusions

This is the first inter-rater reliability study of the Hazelbaker Assessment Tool for Lingual Frenulum Function. (The recent study by Ricke and colleagues compared inter-rater reliability only on the first nine infants assessed [18]).

We found that the HATLFF has a high reliability in recommendation for frenotomy in a study of infants with tongue-tie and control infants. The two assessors had a high degree of agreement in each of the Appearance items and the first three Function items, however there was a lack of agreement between the assessors on each of the four Function items related to infant sucking. We found that it appears that the Function Score can be more simply assessed using only the first three function items (ie not scoring the sucking items).

Further research in this area is needed [19]. Currently, there are a number of other studies underway, for example in Canada, clinicians are developing a simpler tool to assess tongue-tie in breastfed infants [20].

## Competing interests

The authors declare that they have no competing interests.

## Authors' contributions

LHA designed, conducted and analysed the study and drafted the manuscript. JPJ participated in the design and conduct of the study. SMD assisted in study design and analysis. All authors read and approved the final manuscript.
